# Incidence, Microbiology, and Outcomes in Patients Hospitalized With Infective Endocarditis

**DOI:** 10.1161/CIRCULATIONAHA.119.044913

**Published:** 2020-05-15

**Authors:** Anoop S.V. Shah, David A. McAllister, Peter Gallacher, Federica Astengo, Jesús Alberto Rodríguez Pérez, Jennifer Hall, Kuan Ken Lee, Rong Bing, Atul Anand, Dilip Nathwani, Nicholas L. Mills, David E. Newby, Charis Marwick, Nicholas L. Cruden

**Affiliations:** 1 British Heart Foundation Centre for Cardiovascular Science (A.S.V.S., P.G., F.A., J.H., K.K.L., R.B., A.A., N.L.M., D.E.N.), University of Edinburgh, United Kingdom.; 2 Usher Institute of Population Health Sciences and Informatics (A.S.V.S., N.L.M.), University of Edinburgh, United Kingdom.; 3 Institute of Health and Wellbeing, University of Glasgow, United Kingdom (D.A.M., J.A.R.P.).; 4 Academic Health Sciences Partnership in Tayside, Ninewells Hospital and Medical School, Dundee, United Kingdom (D.N.).; 5 Population Health and Genomics, School of Medicine, University of Dundee, United Kingdom (C.M.).; 6 Edinburgh Heart Centre, Royal Infirmary of Edinburgh, United Kingdom (N.L.C.).

**Keywords:** antibiotic, antibiotic prophylaxis, incidence, infective endocarditis, mortality, outcomes

## Abstract

**Background:**

Despite improvements in management, infective endocarditis remains associated with high mortality and morbidity. We describe temporal changes in the incidence, microbiology, and outcomes of infective endocarditis and the effect of changes in national antibiotic prophylaxis guidelines on incident infective endocarditis.

**Methods:**

Using a Scotland-wide, individual-level linkage approach, all patients hospitalized with infective endocarditis from 1990 to 2014 were identified and linked to national microbiology, prescribing, and morbidity and mortality datasets. Linked data were used to evaluate trends in the crude and age- and sex-adjusted incidence and outcomes of infective endocarditis hospitalizations. From 2008, microbiology data and associated outcomes adjusted for patient demographics and comorbidity were also analyzed. An interrupted time series analysis was performed to evaluate incidence before and after changes to national antibiotic prophylaxis guidelines.

**Results:**

There were 7638 hospitalizations (65±17 years, 51% females) with infective endocarditis. The estimated crude hospitalization rate increased from 5.3/100 000 (95% CI, 4.8-5.9) to 8.6/100 000 (95% CI, 8.1–9.1) between 1990 and 1995 but remained stable thereafter. There was no change in crude incidence following the 2008 change in antibiotic prophylaxis guidelines (relative risk of change 1.06 [95% CI, 0.94–1.20]). The incidence rate in patients >80 years of age doubled from 1990 to 2014 (17.7/100 000 [95% CI, 13.4–23.3] to 37.9/100 000 [95% CI, 31.5–45.5]). The predicted 1-year age- and comorbidity-adjusted case fatality rate for a 65-year-old patient decreased in women (27.3% [95% CI, 24.6–30.2] to 23.7% [95% CI, 21.1–26.6]) and men (30.7% [95% CI, 27.7–33.8] to 26.8% [95% CI, 24.0–29.7]) from 1990 to 2014. Blood culture data were available from 2008 (n=2267/7638, 30%), with positive blood cultures recorded in 42% (950/2267). Staphylococcus (403/950, 42.4%) and streptococcus (337/950, 35.5%) species were most common. *Staphylococcus aureus* and enterococcus had the highest 1-year mortality (adjusted odds ratio 4.34 [95% CI, 3.12–6.05] and 3.41 [95% CI, 2.04–5.70], respectively).

**Conclusions:**

Despite changes in antibiotic prophylaxis guidelines, the crude incidence of infective endocarditis has remained stable. However, the incidence rate has doubled in the elderly. Positive blood cultures were observed in less than half of patients, with *Staphylococcus aureus* and enterococcus bacteremia associated with worse outcomes.

## Abstract 2

Supplemental Digital Content is available in the text.

**Clinical Perspective**

**What Is New?**

- Several studies have recently evaluated the changing epidemiology of infective endocarditis before and after guideline recommendations. These studies have predominantly studied the incidence, rather than the outcomes or microbiology, of infective endocarditis.
- Using a national, individual patient-level linkage approach, we describe the changing age- and sex-stratified incidence and outcomes of infective endocarditis in Scotland between 1990 and 2014.
- We further describe temporal changes in patient characteristics and microbiology based on positive blood cultures associated with infective endocarditis.

**What Are the Clinical Implications?**

- The crude incidence rate of infective endocarditis hospitalizations increased from 1990 to 1995 but has remained relatively static thereafter with both short- and long-term adjusted case fatality rates showing a steady decrease over the past 25 years.
- The majority of patients with endocarditis in our cohort did not have positive blood cultures and in those with positive microbiology, staphylococcus and enterococcus conferred the highest risk for all-cause mortality.
- Changes in guidelines regarding antibiotic prophylaxis in the United Kingdom have not resulted in a significant change in incident cases of infective endocarditis.

Despite recent improvements in management, infective endocarditis remains associated with high morbidity and mortality.^1,2^ Over the past few decades, several factors have affected both the incidence and outcomes of infective endocarditis. The population at risk of infective endocarditis has increased because of changes in population demographics, a rise in the use of implantable cardiac devices, an increase in the number of patients undergoing hemodialysis for end-stage renal failure, and a greater number of patients with congenital heart disease surviving to adulthood.^3^ Changes in national guidelines regarding the use of antibiotic prophylaxis for prevention of infective endocarditis^4,5^ have also been implicated in the apparent increase in the incidence of infective endocarditis.

Several studies have recently evaluated the changing epidemiology of infective endocarditis, predominantly incidence,^6^ before and after guideline recommendations.^4,7–10^ Fewer studies have evaluated microbiological causes and associated outcomes of endocarditis.^11,12^ Using a national linkage approach, we describe the changing age- and sex-stratified incidence and outcomes of infective endocarditis in Scotland over the past 25 years and the effect of changes in national guidelines on antibiotic prophylaxis. With the availability of a national microbiology surveillance registry from 2008 onward, we also describe the microbiology of infective endocarditis on the basis of positive blood culture data in a subgroup of infective endocarditis hospitalizations between 2008 and 2014.

## Methods

The data, analytic methods, and study materials will not be made available to other researchers for purposes of reproducing the results or replicating the procedure. However, these individual-level data are available through application to the National Health Service Public Health Scotland. Access to the data were approved by the National Health Service Scotland Public Benefit and Privacy Panel and in accordance with the Declaration of Helsinki. As the analysis used routinely collected and anonymized data, individual patient consent was not sought.

### Study Design and Data Sources

We conducted a consecutive retrospective individual patient linkage study across multiple national databases (Figure I in the Data Supplement). In brief, Scottish hospital discharge codes were used to identify patients hospitalized with infective endocarditis. All episodes for patients ≥20 years of age admitted between January 1, 1990, and December 31, 2014, identified as infective endocarditis were linked to the national hospitalization register (Scottish Morbidity Record 01) in Scotland. Incident cases of infective endocarditis identified from the national hospitalization register between January 1, 2008, and December 31, 2014, were also linked to positive blood culture data derived from individual patients derived through linkage with the national microbiology surveillance database (Electronic Communication of Surveillance in Scotland; Text I in the Data Supplement).

### Participants

Incident episodes of infective endocarditis were identified from hospital inpatient records using the *International Classification of Diseases 9 and 10* codes (Text II in the Data Supplement). On the basis of our validation exercise, only patients with a diagnostic code for endocarditis in the first 2 positions were included (Text III in the Data Supplement). A 5-year look-back period minimized the risk of recurrent episodes of infective endocarditis from being misclassified as incident cases (Text IV in the Data Supplement).

### Covariates

For each patient hospitalization, we extracted age at hospitalization, sex, comorbidity, and socioeconomic status. Socioeconomic status was assessed through a national area-based measure of deprivation, the Scottish Index of Multiple Deprivation (SIMD). The SIMD is measured in quintiles with the fifth quintile being least deprived. In brief, SIMD identifies small geographical regions (each region related to postal [zip] code and corresponding to approximately 750 residents) of material deprivation based on information derived from 7 domains (income; employment; health; education, skills and training; geographic access to services; crime; and housing).^13^ Scores from each domain (which are themselves weighted according to their relative importance) are combined into an overall score, which allows that area to be ranked. Scores and ranks based on SIMD have been used extensively in published epidemiological research from Scotland,^14,15^ including guiding primary prevention in Scotland.^16^ For our study, every patient enrolled in our cohort was assigned a SIMD quintile on the basis of their individual SIMD rank at the time of their index admission.

Patient comorbidities were defined using established *International Classification of Diseases* codes on the basis of previous hospitalizations and procedures using a 5-year look-back period. For every case of infective endocarditis during the 25-year period from 1990 until 2014, we extracted the following comorbidity data: (history of) myocardial infarction, cerebrovascular disease, heart failure hospitalization, implanted cardiac device, and cardiothoracic surgery (Text II in the Data Supplement). From 2009 onward, prescribing data were used to provide additional comorbidity classifications on the basis of dispensed drugs (Text V in the Data Supplement).

### Statistical Analysis

Baseline characteristics of all hospitalizations were summarized by 5 calendar year groups from 1990 to 2014 and in single calendar year groups from 2008 onward. Baseline characteristics were also summarized by blood culture status. Incident hospitalizations were summed by calendar year, age, and sex. Midyear population estimates for the general population in Scotland, stratified by age and sex, were obtained from the National Records of Scotland. For people without incident infective endocarditis, person-time not at risk (attributable to incident infective endocarditis events) was also summed by the same stratifying variables. Within the levels of these stratifying variables, person-time for people with no infective endocarditis was obtained by subtracting the infective endocarditis person-time from the midyear population estimates (Text IV in the Data Supplement). Outcomes consisted of mortality, stroke, heart failure hospitalization, and valve surgery at 30-days and 1-year following the index presentation.

### Modeling

Generalized additive models were used to estimate trends in incidence and outcomes of infective endocarditis. For incidence rates, log-link and Poisson error distribution were used with a scaling factor (quasi-Poisson) to allow for overdispersion. Incidence rates per year were generated for the whole population and stratified by sex and age. For outcomes, 1-year mortality was the primary outcome and year of admission as the primary explanatory variable adjusted for age, sex, comorbidity, and deprivation. For both estimation of trends in the incidence rates and outcomes of infective endocarditis, models were fitted using nonparametric smooth terms (penalized thin plate regression splines) for the year of admission. For the analysis of the association between 30-day and 1-year mortality and microbiology, logistic regression models were constructed adjusted for age, sex, deprivation, and comorbidity.

An interrupted time series analysis^17^ was used to evaluate the incident rates of infective endocarditis before (2001–2007) and after (2008–2014) the introduction of new antibiotic prophylaxis guidelines from the National Institute of Health and Care Excellence (Text VI in the Data Supplement).^18^ A sensitivity analysis was also performed to evaluate the effect of restricting the cohort to the first diagnostic coding position. Statistical analysis was performed in R, version 3.5.1 (Vienna, Austria).

## Results

Across 7513 individual patients, there were 7638 hospitalizations (mean age 65±17 years, 51% women) with incident infective endocarditis from 1990 to 2014 in Scotland (Table 1; Table I in the Data Supplement).

**Table 1.**
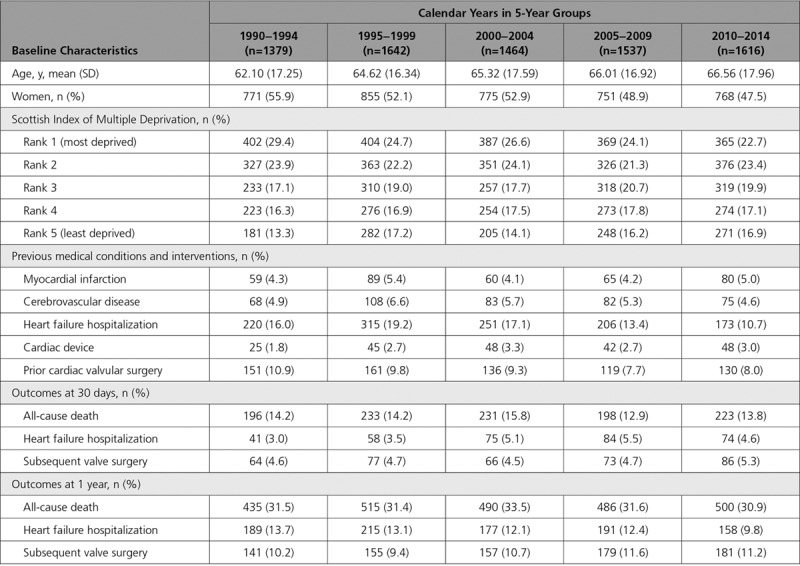
Baseline Characteristics and Outcomes in Patients Hospitalized With Infective Endocarditis, Stratified by 5-Year Calendar Groups

**Figure 1.**
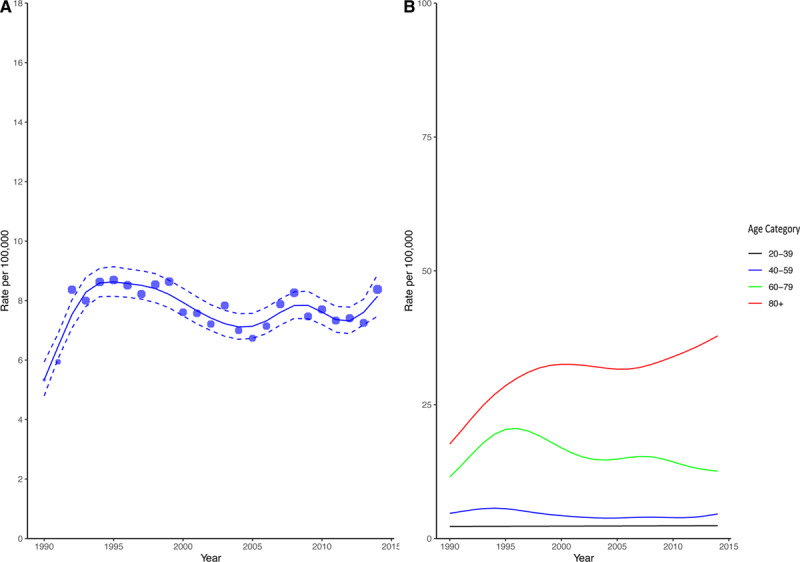
**Estimated incidence rate per 100 000 in the population (A) and stratified by age groups (B).** Blue circles in **A** represent the absolute crude rates with the size of the circles proportional to the absolute count. The solid blue line represents the estimated incident rate from generalized additive modeling using the Poisson distribution. The dashed blue lines represent the corresponding upper and lower 95% CI bounds.

The estimated crude rate of hospitalization increased from 5.3 per 100 000 (95% CI, 4.8–5.9) to 8.6 per 100 000 (95% CI, 8.1–9.1) between 1990 and 1995 but remained stable thereafter with the incident rate in 2014 of 8.1 per 100 000 (95% CI, 7.5–8.9) (Figure 1A; Table II in the Data Supplement). Similar relative changes were seen in the incidence of infective endocarditis in our sensitivity analysis restricting the cohort to the first diagnostic code position (Figure II in the Data Supplement). Estimated rates comparing men and women also appeared similar up until 2003 but appeared to diverge thereafter (Figure III in the Data Supplement; Table II in the Data Supplement).

**Figure 2.**
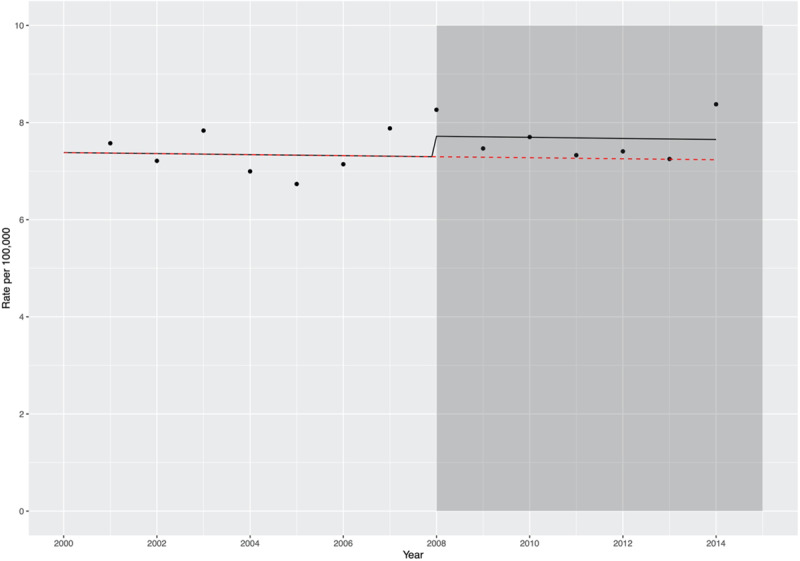
**Plot showing the observed incident rates per 100 000 by year of hospitalization (black dots), in relation to the introduction of national antibiotic prophylaxis guidelines.** The shaded gray box indicates introduction of National Institute of Health and Care Excellence antibiotic prophylaxis guidelines. The black line shows the predicted incident rate using the model described in Text VI in the Data Supplement, incorporating the change in guidelines from 2008 onward. The overlying red line shows the predicted incident rate assuming the counterfactual of no change in antibiotic prophylaxis guidelines in 2008.

When stratified by age, patients >80 years showed a marked increase in the incidence of infective endocarditis rising from 17.7 per 100 000 (95% CI, 13.4–23.3) in 1990 to 37.9 per 100 000 (95% CI, 31.5–45.5) in 2014 (Figure 1B; Table III in the Data Supplement). In contrast, in the 60- to 79-year age group, the estimated rate was 11.5 per 100 000 (95% CI, 10.1–13.2) in 1990 peaking at 20.6 per 100 000 (95% CI, 19.2–22.0) in 1996 before steadily decreasing to 12.6 per 100 000 (95% CI, 11.1–14.3) in 2014. In the younger age groups, the incident rates of endocarditis appeared relatively unchanged (Figure 1B; Table III in the Data Supplement). There was no change in the incident rate of infective endocarditis following implementation of National Institute of Health and Care Excellence guidelines on antibiotic prophylaxis (relative risk of change, 1.06 [95% CI, 0.94–1.20], *P*=0.420; Figure 2).

During the study period, 32% (2426/7638) of patients admitted to hospital with infective endocarditis died within 1 year of admission. Both age and sex influenced 1-year mortality, adjusted for deprivation and comorbidity (Table IV in the Data Supplement). Age-adjusted and sex-stratified predicted case fatality rates are shown in Figure IV and Table V in the Data Supplement. For a 65-year-old female, the predicted risk of 1-year mortality reduced from 27.3% in 1990 (95% CI, 24.6–30.2) to 23.7% (95% CI, 21.0–26.6) in 2014. Similarly, for a 65-year-old male, the risk of 1-year death fell from 30.7% (95% CI, 27.7–33.8) to 26.8% (95% CI, 24.0–29.7). History of cerebrovascular disease (adjusted odds ratio [OR], 1.28 [95% CI, 1.07–1.49]), heart failure hospitalization (OR, 2.09 [95% CI, 1.96–2.22]), and deprivation (OR, 0.92 [95% CI, 0.88–0.95] per unit increment in rank; rank 1 assigned as being most deprived and rank 5 as least deprived) were also associated with a higher risk of death at 1 year.

Data on incident cases of infective endocarditis were available from 1990 to 2014. However, positive blood culture data were also available from 2008 until 2014. As such, the population with blood culture microbiology linkage consisted of 30% (2267/7638) of all hospitalizations (Table 2). Positive blood cultures were recorded in 42% (950/2267) of hospitalizations with the majority of the remainder being culture negative (defined as individuals in whom blood cultures yielded no organism or in whom no cultures were performed).

**Table 2.**
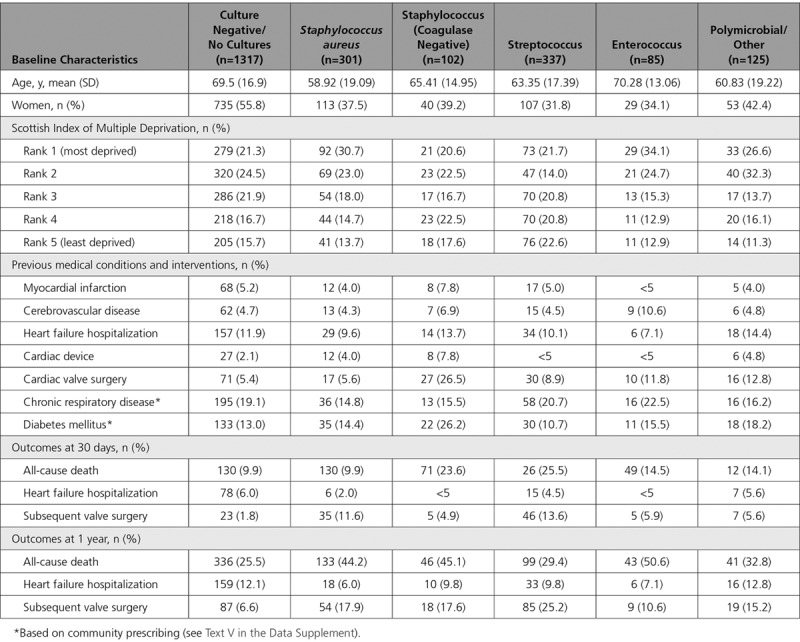
Baseline Characteristics and Outcomes in Patients Hospitalized With Infective Endocarditis, by Blood Culture Status and Organism Identified

From our validation exercise, 12.5% of patients with a clinical diagnosis of infective endocarditis did not have blood cultures taken (Text III in the Data Supplement). At a national level, we could link patient level data to positive microbiology only using the Electronic Communication of Surveillance in Scotland registry. As such, it was neither possible to determine what proportion of the remaining 1317 cases were associated with sterile blood cultures nor possible to determine what proportion did not have blood cultures performed. However, extrapolating from our validation data, where 87.5% of all cases had blood cultures performed, we assume that the majority of the 1317 cases also had blood cultures performed with a significant proportion yielding no growth.

**Figure 3.**
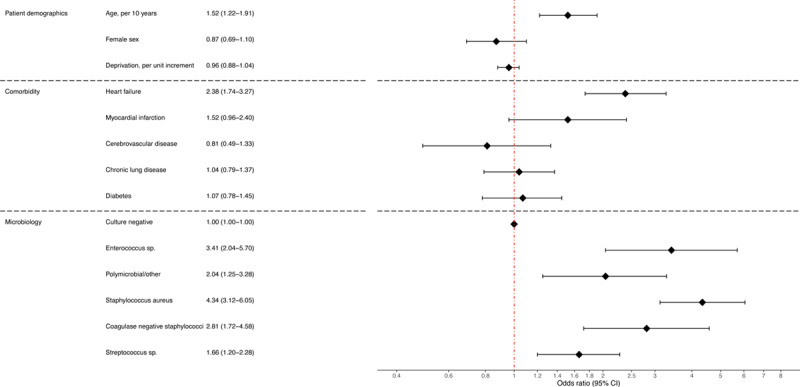
**Forest plot showing odds ratio from logistic regression evaluating the association between all-cause mortality at 1 year and patient demographics, comorbidity, and microbiology.**

Staphylococcus (403/950, 42.4%), streptococcus (337/950, 35.5%), and enterococcus (85/950, 8.9%) were the most common organisms identified (Figure V in the Data Supplement). The majority of staphylococci were *Staphylococcus aureus* (301/403, 74.7%). Across the years, positive microbiology rate increased from 34.7% in 2008 to 45.4% in 2014 (Figure VI in the Data Supplement). Several factors were associated with 30-day and 1-year mortality (Figure VII in the Data Supplement and Figure 3, respectively). Compared with patients without positive blood cultures, those with *Staphylococcus aureus* and enterococcus were at the highest risk of 1-year mortality (OR, 4.34 [95% CI, 3.12–6.05] and OR, 3.41 [95% CI, 2.04–5.70], respectively).

In our sensitivity analysis restricting the cohort to the first diagnostic code position, positive blood cultures were identified in 63% (753/1195) of patients (Figure VIII in the Data Supplement). Similar to the primary analysis, those with *Staphylococcus aureus* and enterococcus were at the highest risk of 1-year mortality (OR, 2.23 [95% CI, 1.47–3.39] and OR, 1.89 [95% CI, 1.05 – 3.42], respectively), compared with patients without positive blood cultures (Figure IX in the Data Supplement).

## Discussion

In this nationwide population cohort study using individual patient-level data linkage, we make several important observations. First, although the estimated overall crude incidence of infective endocarditis has remained relatively stable from 1995 onward, when stratified by age, there was a 2-fold increase in incidence in the elderly but either decreasing or static rates in younger patients. Second, the adjusted case fatality rate after endocarditis remains high but has declined over the past 25 years. After adjustment for age and comorbidity, men had overall higher case fatality rates than women. Third, fewer than half of all patients had positive blood cultures, with the overall positive blood culture rate increasing from 35% in 2008 to slightly less than half in 2014. Where a causative organism was identified, staphylococcus and streptococcus were the most common species. Fourth, compared to patients with no positive blood cultures patients with *Staphylococcus aureus* or enterococcus bacteremia had the highest risk of death. Last, and perhaps of most clinical relevance, our analysis has shown that the 2008 change in antibiotic prophylaxis guidelines in the United Kingdom has not resulted in a significant rise in incident cases of infective endocarditis.

Population-based studies in endocarditis remain scarce with the majority of epidemiology extrapolated from hospital-based cohorts or cross-sectional studies.^19,20^ Our dataset included cases of infective endocarditis over the past 25 years. Whereas we only had positive blood culture data for a fifth of this period, evaluating the overall period allowed us to investigate long-term trends in incidence of and outcomes after hospitalization with infective endocarditis. With the exception of recent large population-based time-series analyses evaluating the temporal incidence of endocarditis in England^4,21^ and the United States,^7,22^ the majority of studies are limited by sample size, consisting of <1000 patients.^20^ All of these studies are further limited by incomplete characterization of participant demographics and characteristics, including comorbidity, case-fatality rates, and microbiology. Using an established national linkage approach in Scotland,^23,24^ we identified >7000 hospitalizations with a diagnosis of infective endocarditis and provide detailed individual patient-level information on baseline demographics, comorbidity burden, associated microbiology data, and subsequent case fatality rates.

As such, there are several strengths to our study. First, our approach ensured complete follow-up in those patients who remained resident in Scotland during the study period. Indeed, similar linkages have already been used to deliver randomized clinical trials^23,25^ and cohort studies^24^ in Scotland. Second, our cohort consisted of consecutive patients hospitalized with a diagnostic code for infective endocarditis, avoiding selection bias and ensuring that our study population was representative. Third, unlike previous administrative data assets using diagnostic coding data to determine culture status,^7,22^ which may not have been restricted to blood stream infections, we have used primary patient-level data from Scottish microbiology laboratories to ensure accurate recording of positive blood culture status. Fourth, using source clinical data, we validated the accuracy of the diagnostic coding including code position and microbiology data using data from local microbiology laboratory information systems (ensuring consistency with the national microbiology surveillance systems across Scotland).

Three relatively recent guideline updates have emerged from the National Institute of Health and Care Excellence^18^ in the United Kingdom, the American Heart Association,^26^ and the European Society of Cardiology.^2^ These guidelines have recommended either complete or partial cessation of antibiotic prophylaxis in patients at moderate or high risk of infective endocarditis. Subsequent analyses evaluating the incidence of infective endocarditis have shown contrasting results. In England, a significant reduction in antibiotic prescriptions and an apparent rise in the incidence of endocarditis was observed following the introduction of changes to National Institute of Health and Care Excellence guidelines.^2,4^ Similar results were noted in a US-based population comparing incidence before and after changes to antibiotic prophylaxis guidelines in 2007.^7^ In contrast, 3 studies from the United States showed no increase in incidence following changes to antibiotic prophylaxis guidelines.^8,27,28^ These studies, however, were either limited in terms of cohort size^8^ or evaluated incident rates over a shorter time frame following the change in guidelines.^27^ Furthermore, small but well-characterized populations in the United States^29^ and France^10^ have also reported static incident rates. In our study, the crude incident rate of infective endocarditis increased in Scotland from 1990 until 1995 but remained relatively static thereafter, suggesting that changes to clinical guidelines regarding antibiotic prophylaxis were not associated with an adverse effect on the incidence of infective endocarditis in Scotland.

We observed an important interaction between age and rates of endocarditis. The incidence in patients older than 80 years of age increased 2-fold, while remaining static or decreasing in younger populations. This striking increase in the elderly is multifactorial and most likely reflects changes in the incidence of degenerative valvular heart disease, a rise in the number of patients surviving with multiple comorbidities, an increase in the provision of invasive therapeutic interventions, including implantable cardiac devices (pacemakers, defibrillators, closure devices, and percutaneous valve technology)^30^ and hemodialysis,^31^ and more extensive investigations^32^ in frail, elderly patients.

We report important differences in the microbiology of patients with infective endocarditis compared with many studies in the existing literature. In our study, with linkage to a robust national microbiology laboratory blood culture dataset, no causative organism was identified in the majority (57%) of patients. In the published literature, culture-negative endocarditis varied from 15% to 60% across both hospital-based cohorts^20,33^ and population registries that are based primarily on US populations.^7,22,34^ Several reasons might explain our low culture-positive rates. First, we defined infective endocarditis using diagnostic coding. Although this is more sensitive and eliminated selection bias, it is possible that the lower rates of culture-positive cases reflect lower specificity by including some patients with lower probability of infective endocarditis. We validated the accuracy of the diagnostic coding using an in-depth health record review of nearly 400 cases. Restricting the patient population to the first 2 diagnostic positions gave an overall positive predictive value for definite or probable diagnosis of infective endocarditis of 88%. As such, case ascertainment bias caused by coding error is unlikely to have made a significant contribution. Second, from 2009 to 2012, 3 laboratories did not provide complete microbiology data for national linkage. These laboratories were small and served <1.5% of the Scottish population and would therefore have a negligible effect on the rates of positive blood cultures. Third, across most hospitalized cohorts of patients with infective endocarditis, cases were identified by the attending clinician.^35^ A high culture-positive rate in these cohorts may therefore reflect selection bias toward patients with positive blood cultures.^11^ Across population registry data, the culture-negative rates were higher^7,22^ with similar rates to those observed in Scotland.^34^ Fifth, across both population registries^7,22^ and some hospital-based cohorts,^36^ culture status was not restricted to blood cultures but also included tissue cultures including valves and serological tests, invariably increasing the rates of culture-positive diagnosis.^36^ It is reassuring that, where positive blood cultures were obtained in our cohort, streptococcus and staphylococcus were the most commonly identified pathogens, consistent with the wider literature.^37,38^ Last, we noticed a relative rise of 16% in the proportion of patients with culture-positive infective endocarditis from 2008 to 2014. This observation likely reflects more judicious timing of administration of antibiotic treatment and ensuring blood cultures are taken prior to initiation of therapy; clinical practice that has been emphasized in recent international guidelines.^39^

Across the study cohort, crude temporal mortality rates at 1-year remained stable, ranging from 27% to 33%. However, after adjustment, we observed a steady reduction in mortality. A recent large US-based study showed a similar relationship with decreasing mortality over a similar time period^22^ while others have shown little or no change in case fatality.^4,40^ Positive microbiology was independently associated with poorer outcomes. *Staphylococcus aureus* bacteremia was independently associated with a 4-fold increased mortality rate at 30-days and 1-year. In contrast, the magnitude of association for enterococcus bacteremia changed from a 2-fold increased risk at 30-days to 3-fold increased risk at 1 year. This observation likely reflects a frailer and older population that is more susceptible to enterococcus bacteremia^41,42^ and at higher risk of medium- to long-term mortality.^43–45^

## Limitations

There are several limitations to our study. First, index cases and comorbidity were defined using administrative datasets, which are subject to inaccurate coding. However, we improved coding specificity for infective endocarditis using data from our validation work. To ensure a reasonable balance between specificity and sensitivity, we included all hospitalizations with a diagnostic code appearing in the first 2 (of 6) positions only. Restricting the population to the first diagnostic position markedly reduced the sensitivity for infective endocarditis in previous literature.^22^ Second, for our validation exercise, the diagnosis of infective endocarditis was on the basis of a clinician documented diagnosis of infective endocarditis. Although we acknowledge that the Modified Duke Criteria represent the gold standard for defining cases of infective endocarditis,^2^ unfortunately the majority of clinicians did not reference these criteria in clinical notes entered on electronic patient records. For example, the presence or absence of physical examination findings relevant to the minor criteria (eg, vascular or immunologic phenomena) were frequently not documented. After careful consideration of the impact of these missing data, and the potential for introducing significant bias, our research team elected to use a more pragmatic approach with true cases of infective endocarditis defined as those clinically documented and treated as infected endocarditis. Third, we could not differentiate patients with blood culture-negative infective endocarditis from those patients in whom blood cultures were not performed. From our validation work, we suspect that just more than 10% of all patients will fall into the latter category. Fourth, while we have attempted to address the majority of confounders, residual unmeasured confounding may have affected the trends observed in case fatality rates and the associations evaluating microbiology and risk of mortality.

## Conclusions

We report important temporal changes in patients with infective endocarditis in the Scottish population over the past 25 years. The crude incidence of infective endocarditis increased from 1990 to 1995 but has remained relatively static thereafter. It is of note that the incidence has increased 2-fold in the elderly. Both short- and long-term adjusted case fatality rates of infective endocarditis have shown a steady decrease over the past 25 years. Last, and of most interest, the majority of patients with infective endocarditis in our cohort did not have positive blood cultures. In those patients with positive microbiology, staphylococcus and enterococcus conferred the highest risk of all-cause mortality. Our data highlight that infective endocarditis remains a lethal condition, especially in the elderly. We also demonstrate the importance of microbiology data for prognostication, not only for in-hospital mortality but also for medium-term outcomes. As such, our data further support the multidisciplinary integration of cardiology, microbiology and infectious disease teams as advocated by international guidelines to optimize diagnosis and patient care.^2^

## Sources of Funding

This work was funded by the British Heart Foundation through an Intermediate Clinical Research Fellowship (FS/19/17/34172), Clinical Research Training Fellowship (FS/18/25/33454), Senior Clinical Research Fellowship (FS/16/14/32023), a Research Excellence Award (RE/18/5/34216), and Academy of Medical Sciences Starter Grants for Clinical Lecturers Scheme. Dr McAllister is supported by a Wellcome Trust Intermediate Clinical Fellowship (201492/Z/16/Z), and Dr Newby is supported by the British Heart Foundation (CH/09/002, RG/16/10/32375, RE/18/5/34216) and by a Wellcome Trust Senior Investigator Award (WT103782AIA).

## Disclosures

None.

## Supplemental Materials

Data Supplement Text I–VI

Data Supplement Figures I–IX

Data Supplement Tables I–VII

References 46–48

## Supplementary Material


