# Supplementary material for: Incidence, Microbiology, and Outcomes in Patients Hospitalized With Infective Endocarditis
Source: Circulation. 2020 May 15;141(25):2067–77. doi: 10.1161/CIRCULATIONAHA.119.044913 (PMC7306256; doi:10.1161/CIRCULATIONAHA.119.044913)
Supplement: Supplementary file 1 [file cir-141-2067-s001.pdf]

SUPPLEMENTAL MATERIAL

Incidence, microbiology and outcomes in patient hospitalized with infective endocarditis

Anoop S V Shah, MD PhD,[1,2*] David A. McAllister, MD,[3*] Peter Gallacher, MD,[1]
Federica Astengo, BSc,[1] Jesús Alberto Rodríguez Pérez, BSc,[3] Jennifer Hall, MD, [1]
Kuan Ken Lee, MD,[1] Rong Bing, MD,[1] Atul Anand, MD,[1] Dilip Nathwani, MD,[4]
Nicholas L. Mills, MD PhD,[1,2] David E. Newby, MD PhD,[1] Charis Marwick, MD PhD,[5]
Nicholas L. Cruden MD PhD.[6]

[1] BHF Centre for Cardiovascular Science, University of Edinburgh, Edinburgh, UK.
[2] Usher Institute of Population Health Sciences and Informatics, University of Edinburgh, UK.
[3] Institute of Health and Wellbeing, University of Glasgow, Glasgow, UK.
[4] Academic Health Sciences Partnership in Tayside, Ninewells Hospital and Medical School, Dundee, UK.
[5] Population Health & Genomics, School of Medicine, University of Dundee, Dundee, UK.
[6] Edinburgh Heart Centre, Royal Infirmary of Edinburgh, Edinburgh, UK.

[*]Contributed equally

Corresponding Author:
Dr Anoop S V Shah
BHF/University Centre for Cardiovascular Science
The University of Edinburgh
Edinburgh EH16 4SA
United Kingdom
Telephone: +44 131 242 6515
Fax: +44 131 242 6379
E-mail: Anoop.shah @ed.ac.uk

Supplementary figures

Supplementary figure I**: *Map summarising linkage of national data assets to define the study population and subsequent longitudinal follow up. Nomenclature: Anonymised patient ID- Community Health Index (CHI) number; Community prescriptions and dispensing- Prescribing Information System (PIS); National blood culture repository- Electronic communication of surveillance in Scotland (ECOSS); Scottish hospitalization record- Scottish Morbidity Record (SMR) 01.***

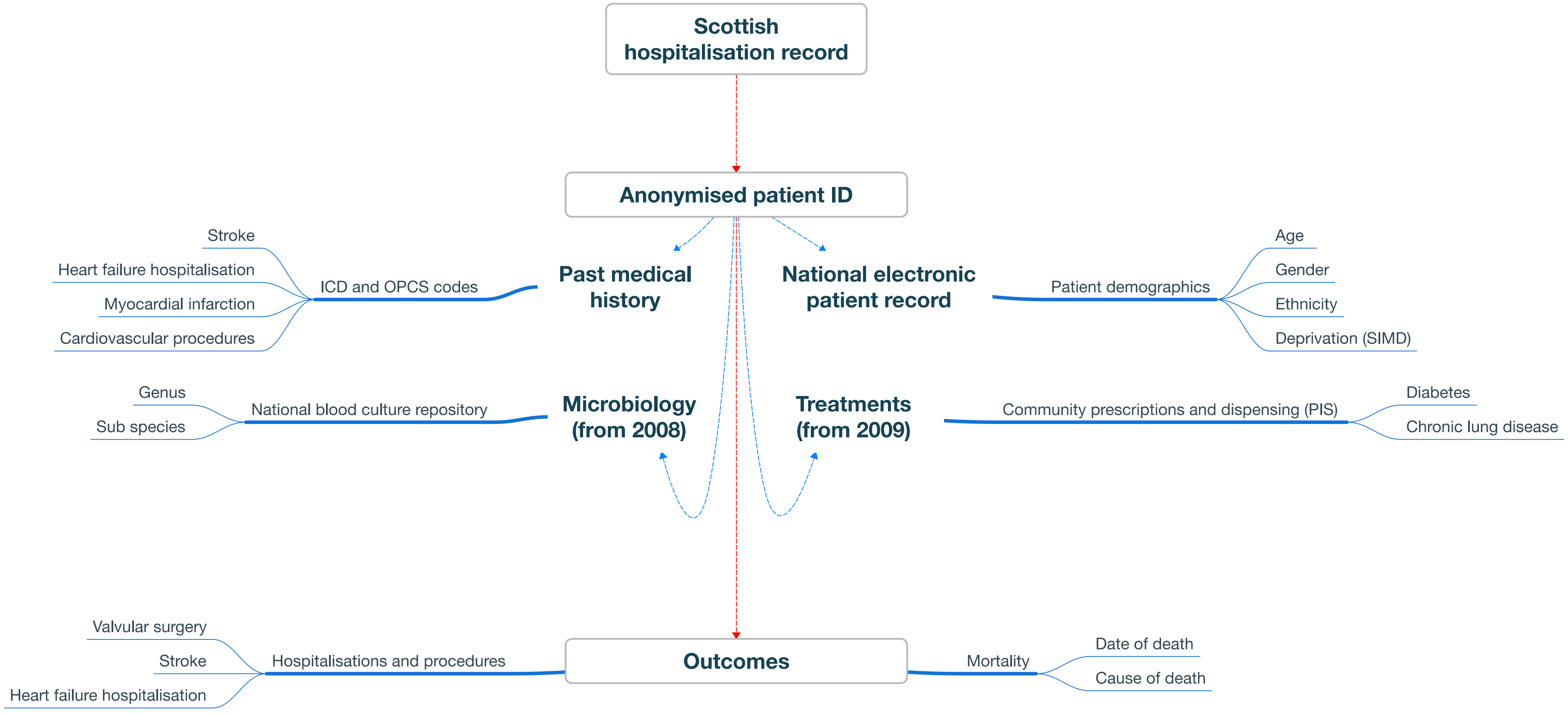

Supplementary figure II: Sensitivity analysis restricting to diagnostic coding in position one (primary diagnosis). Estimated incidence rate per 100,000 in the population. Blue circles represent the absolute crude rates with the size of the circles proportional to the absolute count.

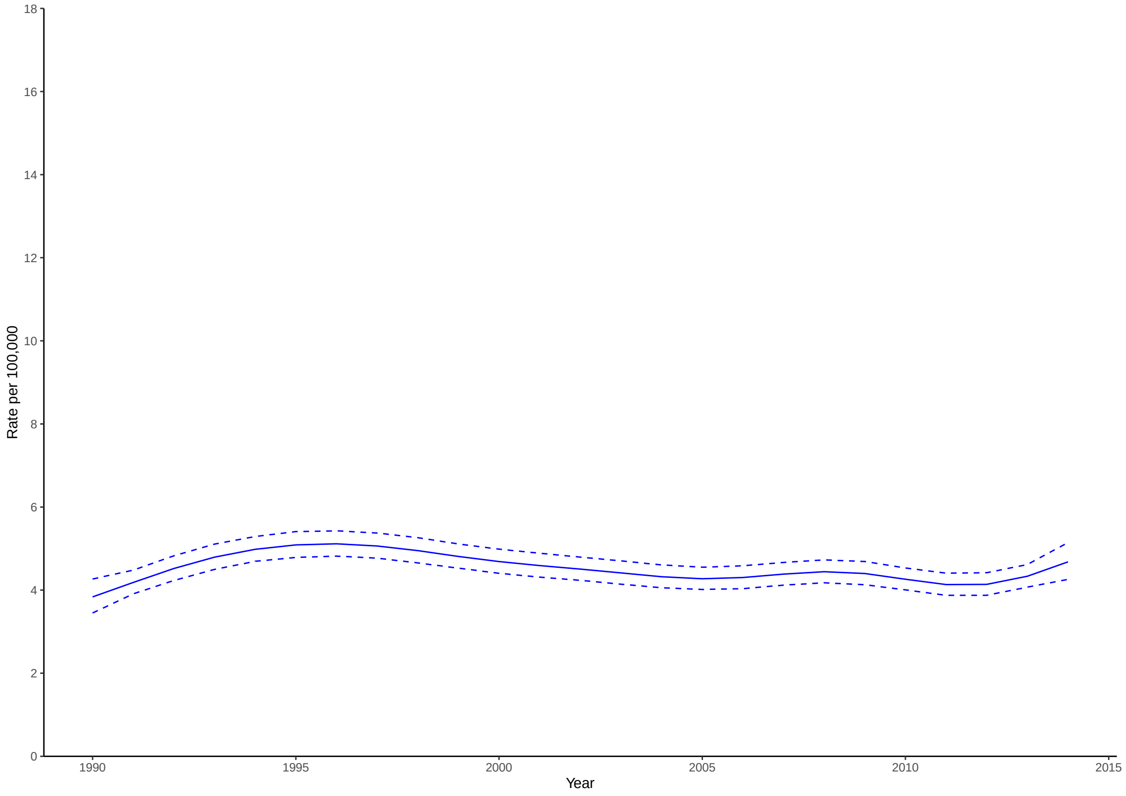

Supplementary figure III: Estimated incidence rate per 100,000 stratified by sex.

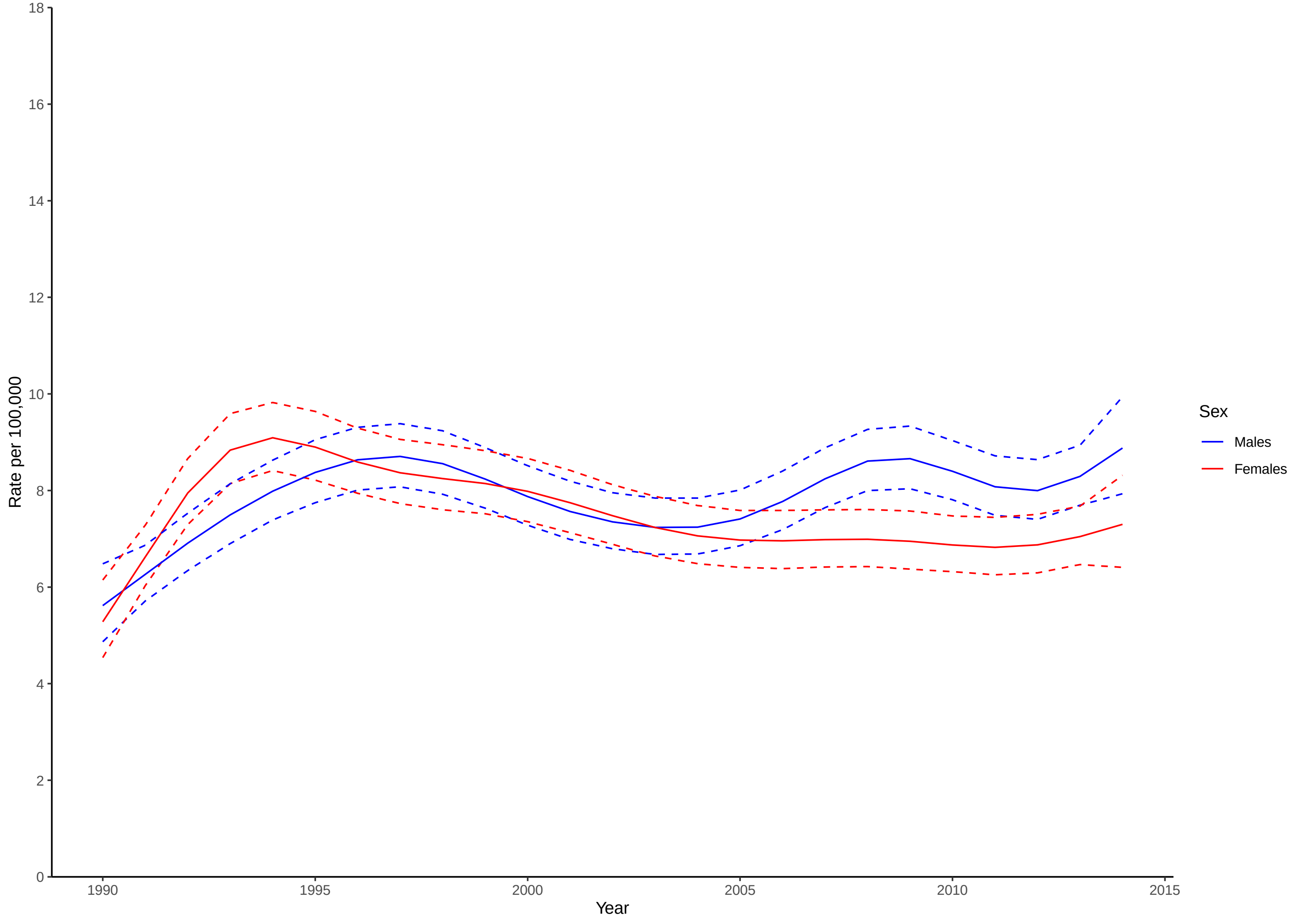

Supplementary figure IV: Predicted one-year mortality following incident endocarditis in men and women.

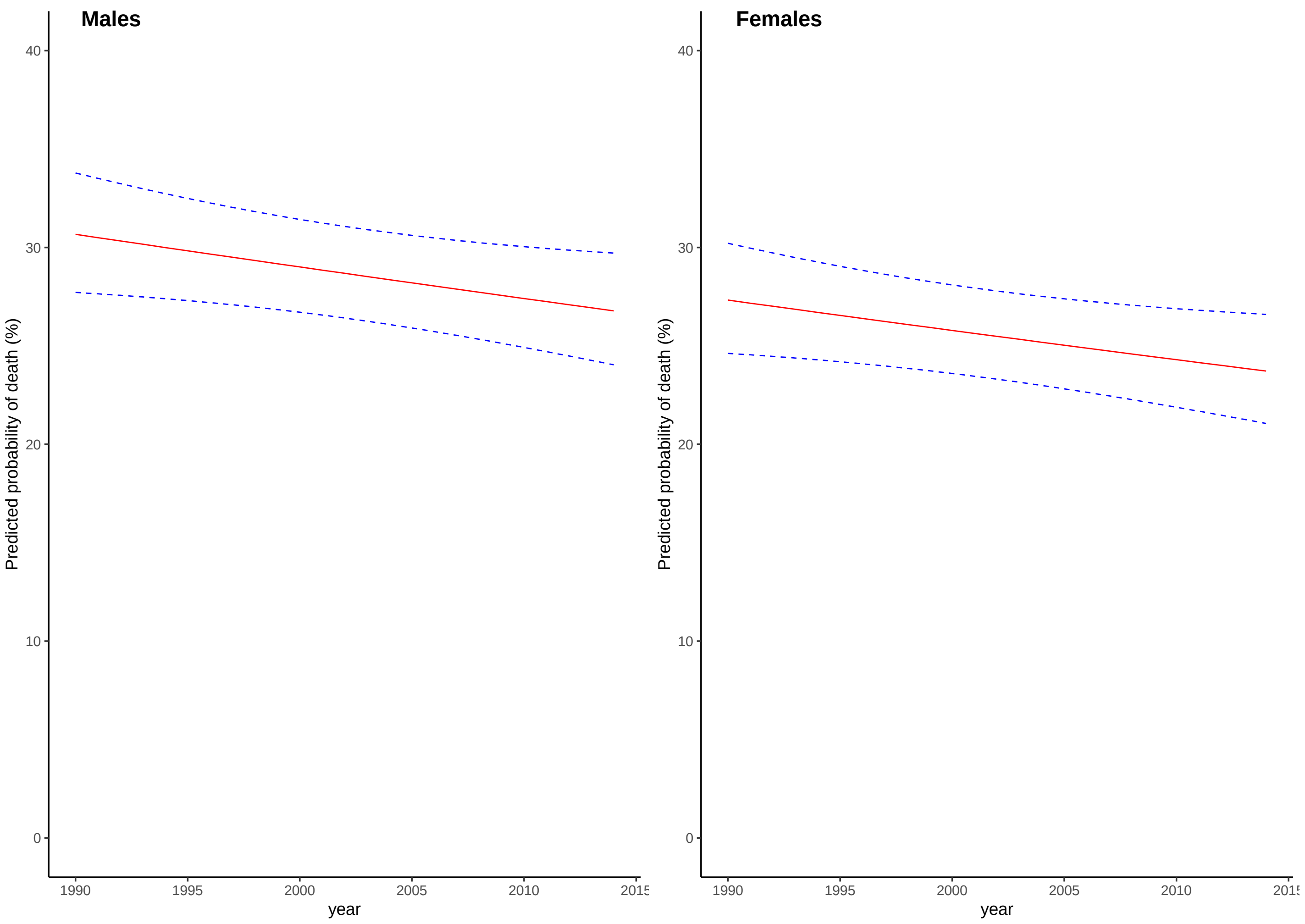

Supplementary figure V: Positive blood culture microbiology stratified by species

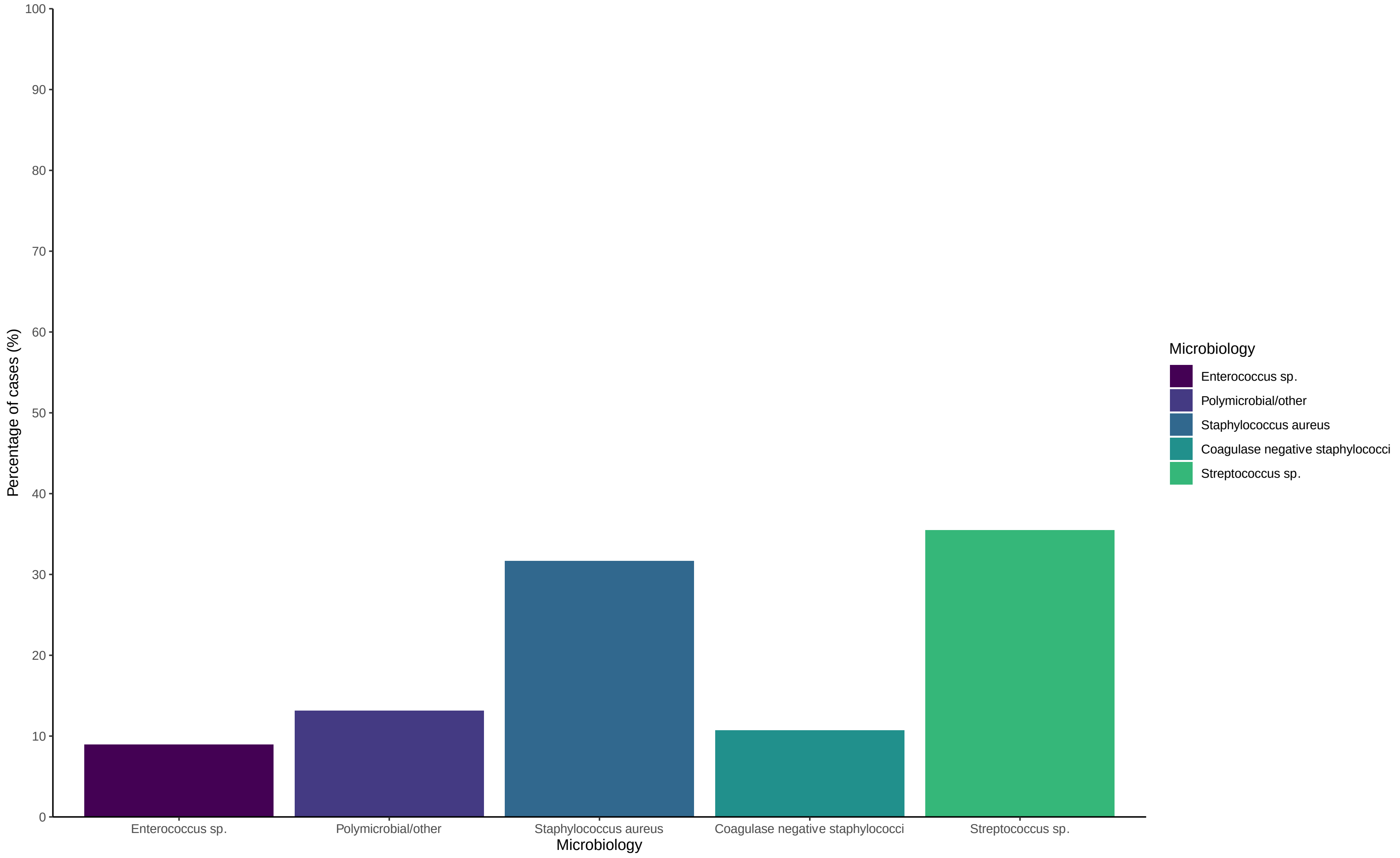

Supplementary figure VI: Stack plot showing microbiology by year

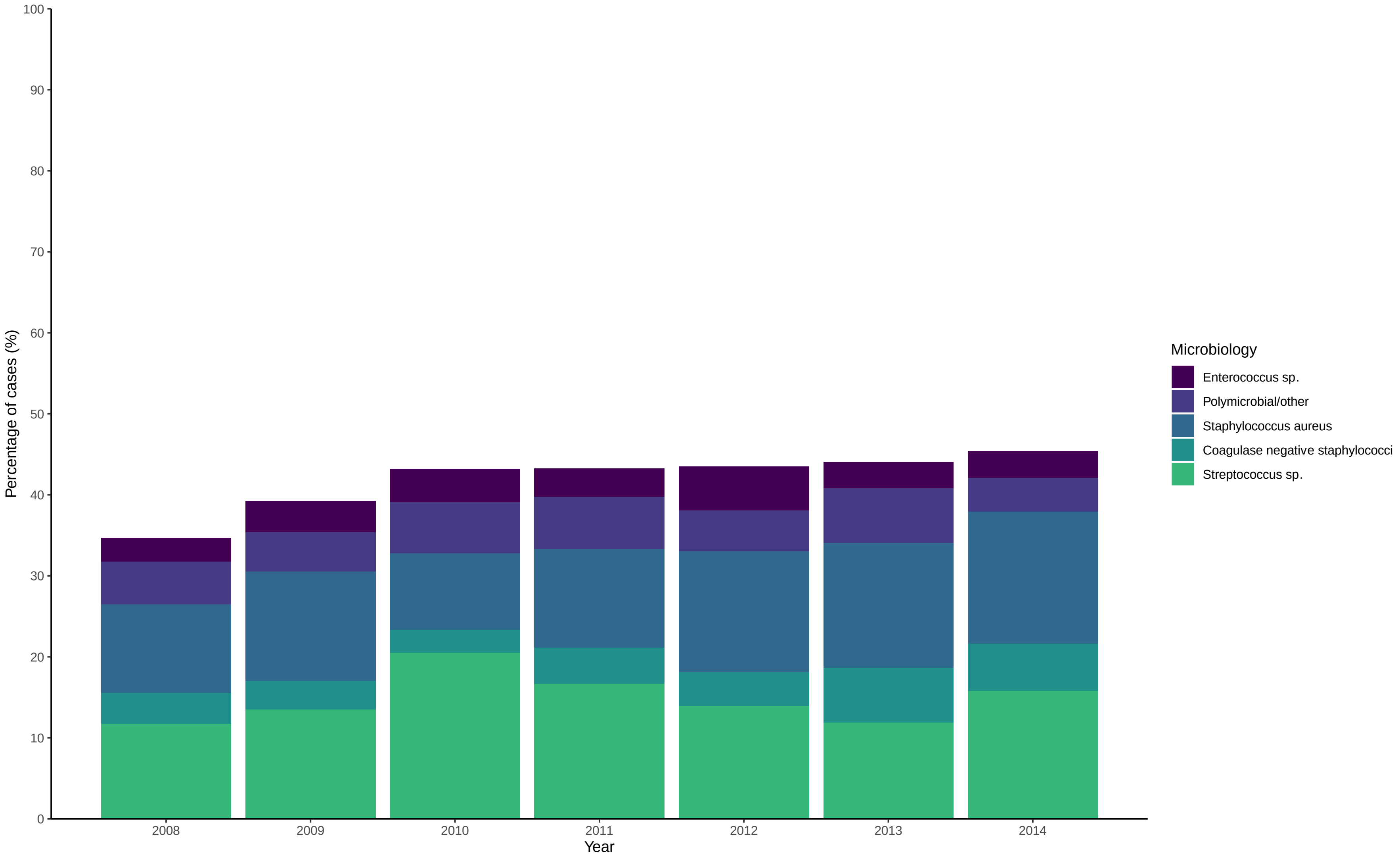

Supplementary figure VII: Forest plot showing association of microbiology and risk of mortality at 30 days

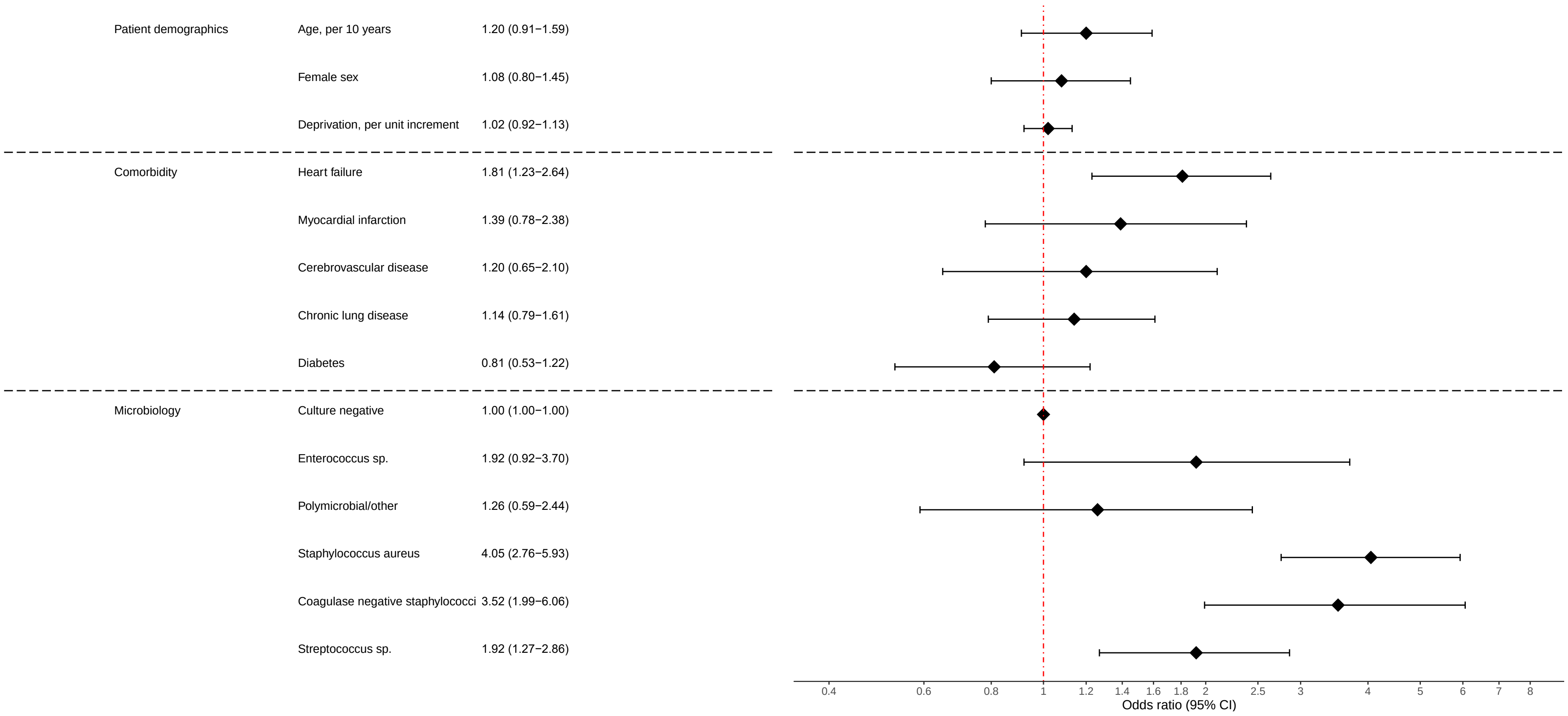

Supplementary figure VIII: Sensitivity analysis restricting diagnostic coding to position one. Microbiology status and organism identified.

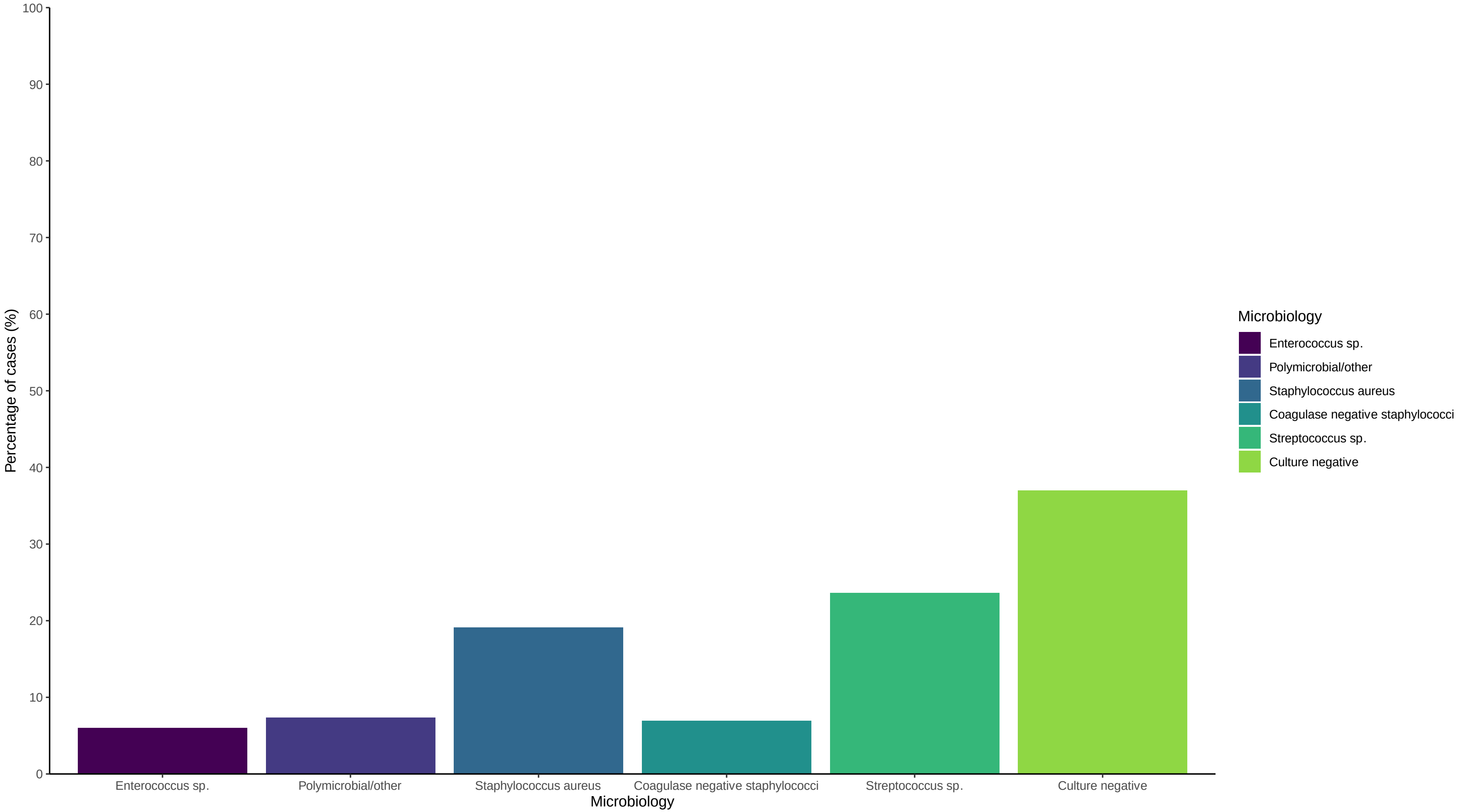

Supplementary figure IX: Sensitivity analysis restricting to diagnostic coding in position one. Forest plot showing association of microbiology and risk of mortality at 1 year

Patient demographics	Age, per 10 years	1.60 (1.19−2.15)
	Female sex	0.93 (0.67−1.29)
	Deprivation, per unit increment	0.95 (0.85−1.06)
Comorbidity	Heart failure	1.66 (1.08−2.55)
	Myocardial infarction	1.50 (0.82−2.75)
	Cerebrovascular disease	0.75 (0.36−1.50)
	Chronic lung disease	1.03 (0.70−1.50)
	Diabetes	1.04 (0.68−1.56)
Microbiology	Culture negative	1.00 (1.00−1.00)
	Enterococcus sp.	1.89 (1.05−3.42)
	Polymicrobial/other	0.94 (0.48−1.78)
	Staphylococcus aureus	2.23 (1.47−3.39)
	Coagulase negative staphylococci	1.63 (0.91−2.89)
	Streptococcus sp.	0.92 (0.61−1.37)

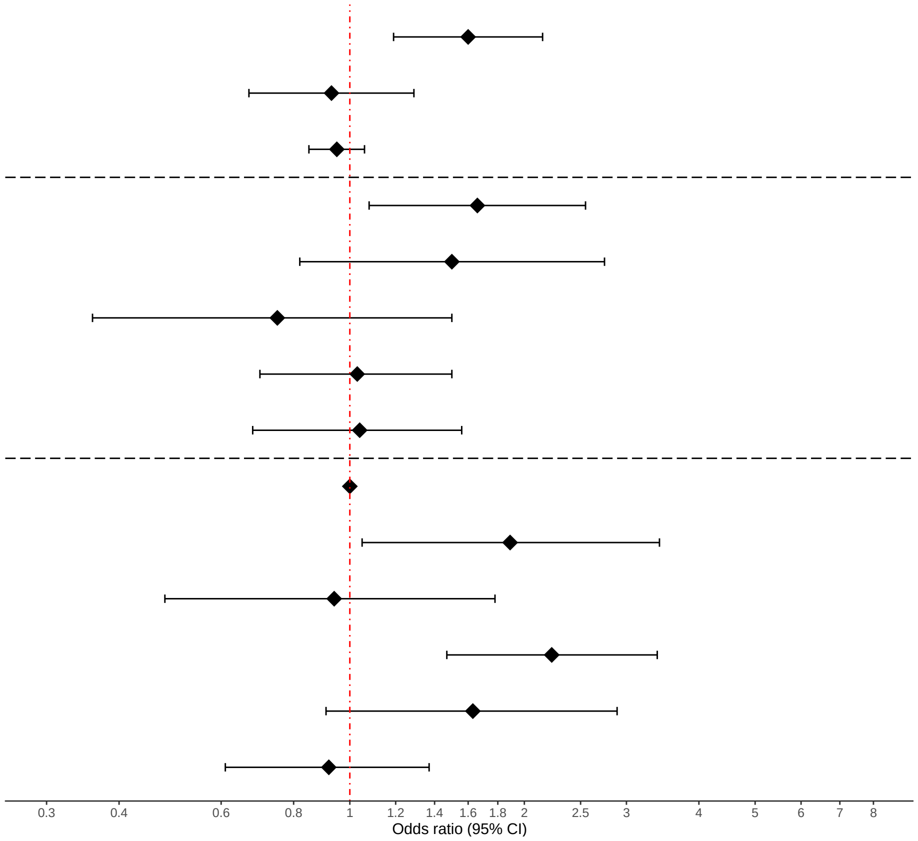

Supplementary text

Supplementary text I: Descriptions and sources of national data assets used for individual patient level linkage

Scottish hospitalization record

Scottish hospitalizations from infective endocarditis were defined from the Scottish morbidity record 01 (SMR01) - General/Acute Inpatient & Day Case. SMR01 is an episode-based patient record relating to all inpatients and day cases discharged from non-obstetric and non-psychiatric specialties. A record is generated when a patient completes an episode of inpatient or day case care. Data collected include patient identifiable and demographic details, episode management details and general clinical information. Currently diagnoses are recorded using the ICD-10 classification and operations are recorded using the OPCS-4 classification. Further information on the national dataset and variables contained is available at https://www.ndc.scot.nhs.uk/Data-Dictionary/SMR-Datasets//Episode-Management/SMR-Record-Type/

Past history, operation codes and demographics were also derived from the Scottish Morbidity Record 01 and linked to incident cases of infective endocarditis. Subsequent hospitalizations based on ICD codes are further linked to incident cases of infective endocarditis identified.

National Records of Scotland (NRS)

The NRS covers all deaths in Scotland with approximately 55,000 deaths registered annually. The National Records of Scotland Death Records are linked with the NHS Scotland Scottish Morbidity Database which links together NHS Scotland inpatient, mental health and cancer registry datasets with the NRS Death Records.

Death status, cause of death and date of death were linked to the patients defined as having incident infective endocarditis.

Further information of the NRS death registry is available at https://www.ndc.scot.nhs.uk/National-Datasets/data.asp?SubID=13

Prescribing Information System (PIS)

The Prescribing Information System (PIS) is the definitive data source for all prescribing relating to all medicines and their costs that are prescribed and dispensed in the community in Scotland. The information is supplied by Practitioner & Counter Fraud Services Division (P&CFS) who is responsible for the processing and pricing of all prescriptions dispensed in Scotland. Primary care physicians write the vast majority of these prescriptions, with the remainder written by other authorised prescribers such as nurses and dentists. Also included in the dataset are prescriptions written in hospitals that are dispensed in the community. Note that prescriptions dispensed within hospitals are not included.

Both the diabetes and chronic lung disease status in our cohort were based on community prescribing data as per ***Supplementary table 5***. Incident cases of endocarditis prescribed either anti-diabetic drugs or drugs for chronic lung disease with one year prior to hospitalization were defined as having the condition. Complete prescribing data was available from 2009.

Further information on the Prescribing Information System operational in Scotland is available at https://www.ndc.scot.nhs.uk/National-Datasets/data.asp?SubID=9

National microbiology register (Electronic Communication of Surveillance in Scotland [ECOSS])

The Scottish microbiology surveillance registry, or '*Electronic Communication of Surveillance in Scotland*' (ECOSS) as it is termed by NHS National Services Scotland, was used in the present study to provide individual patient-level data on positive blood culture results (from diagnostic microbiology laboratories within NHS Scotland health boards and national reference laboratories) related to incident cases of infective endocarditis identified from SMR01 between 2008 and 2014. ECOSS is part of NHS Scotland's Infection Intelligence Platform (IIP),[46,47] which was set-up in response to the UK's antimicrobial resistance (AMR) strategy (2013-2018) with the aim of providing "*better access to and use of surveillance data*".[48]

Data were first collected and recorded within ECOSS in 2007. The dataset is maintained by NHS National Services Scotland on behalf of Health Protection Scotland. ECOSS is updated monthly and, as of 2017, it contained approximately 29 million records of positive microbiology laboratory specimens from across Scotland.[1] It provides data for numerous national clinical and research activities, audit projects and Scottish Government reports, including: the identification of cases of severe infectious disease, infectious disease outbreaks and the evaluation of longer term trends in the incidence of laboratory-reported infections; surveillance of episodes of Clostridium difficile infections, Escherichia coli bacteremia, Staphylococcus aureus bacteremia and surgical site infections.[2] NHS National Services Scotland monitors the completeness and accuracy of ECOSS data through its 'Data Monitoring and Support Service'.[1] Further, NHS National Services Scotland routinely informs data users of any problems affecting the accuracy or assurance of these data.

In the present study, causative organisms were defined as those identified within 90 days on either side of the index admission date. Using this timeframe, 950 cases of infective endocarditis were associated with positive microbiological results.

Polymicrobial status was defined specifically is more than one causative organism was identified on the same culture date. If more than one causative organism was identified on differing dates, then the organism identified closest to the index admission date was assigned as the causative organism.

Near complete blood culture microbiology data were available from 2008. Three small Scottish laboratories did not provide complete data. These laboratories were as follows:

- Shetland (GIL:BAN) – no data for 2009-2011; for 2012 we only received two blood culture reports
- Western Isles (WES:LES) – no data for 2009-2010
- Orkney (ORK:BAL) – 2009 only one blood culture report received; no data for 2010

Overall these laboratories served <1.5% of the Scottish Population and would therefore have a negligible effect on the rates of non-positive blood cultures observed.

More information on the ECOSS data system is available at https://www.hps.scot.nhs.uk/data/

Approvals for use of data

Access to the data was approved by the NHS Scotland Public Benefit and Privacy Panel and in accordance with the Declaration of Helsinki. As the analysis used routinely collected and anonymized data, individual patient consent was not sought.

Supplementary text II: ICD-9 and ICD-10 code identifiers employed to identify cases of infective endocarditis from SMR01 dataset

Co-morbidity	Relevant ICD codes
**Infective endocarditis**	
ICD-9	421.1, 424.91, 424.90, 424.99
ICD-10	I33, I38, I39
**Myocardial infarction**	
ICD-9	413
ICD-10	I21, I22
**Cerebrovascular disease**	
ICD9	430 - 438
ICD10	I60-I69
**Heart failure**	
ICD-9	428
ICD-10	I50
**Valve surgery**	
OPCS	K04 - K12, K14, K17 - K34
**Cardiac device**	
OPCS	K59 - K61

Supplementary text III: Validation of SMR01 ICD-9 and ICD-10 coding for diagnosis and microbiology of infective endocarditis

Electronic hospital records of 396 episodes of suspected infective endocarditis dating from a 5-year period (2014-2018) were manually reviewed by two authors (PG and JH) as part of the validation exercise. The electronic records were reviewed to determine if the diagnosis of infective endocarditis was accurate.

SMR01 diagnostic code position	Positive predictive value, % (95% CI)
Main condition only (1)	97.1 (95.0 to 99.0)
Main condition and second diagnostic code position (1-2)	88.6 (84.7 to 92.5)
Main condition, second and third diagnostic code positions (1-3)	79.6 (75.7 to 83.5)
Any diagnostic code position (1-6)	67.9 (64.0 to 71.8)

Thereafter, electronic hospital records of 200 episodes of suspected infective endocarditis with a diagnostic code position of 1-3 and 168 episodes of suspected infective endocarditis with a diagnostic code position of 1-2 were reviewed in order to determine if blood cultures had been sent within 6 months of the index diagnosis from the admitting hospital.

SMR01 diagnostic code position	Overall blood cultures sent, n (%)
Main condition and second diagnostic code position (1-2)	147 (87.5)
**SMR01 diagnostic code position**	**Organism grown in blood cultures, n (%)**
Main condition and second diagnostic code position (1-2)	89 (52.9)

Clinical definition of infective endocarditis in our validation exercise

Although the Modified Duke Criteria (MDC) represent the 'gold standard' for defining cases of infective endocarditis, we were unable to employ the MDC reliably in our validation exercise. This was partly because the vast majority of clinicians did not reference the MDC in their documentation. Further, the presence or absence of key physical exam findings relevant to the minor criteria (e.g. vascular or immunologic phenomena) were frequently not

documented in patient records. After careful consideration of the impact of these missing data and the potential for introducing significant bias if we persisted with the MDC to define cases of infective endocarditis, our research team elected to employ a more pragmatic approach in the definition of infective endocarditis from electronic clinical records. The definition to define endocarditis has been summarised below.

Definition

True IE: Clinician diagnosis of IE documented in patient notes **and** patient treated as IE.

No IE: No clinician diagnosis of IE documented in patient notes **and/or** patient not treated as IE.

As mentioned above our validation work included all local cases of infective endocarditis from 2014 until 2018 (n=396). Of these, infective endocarditis was the first or second diagnostic code in 67% (264/396) of hospitalizations, and the first diagnostic code in 53% (208/396) of hospitalizations during this period. Confirming infective endocarditis based on the above definition and a diagnostic code position of one or two provided an overall positive predictive value of 88.6%. The table below summarises our validation work stratified by clinician adjudicated diagnosis of infective endocarditis and diagnostic position:

Certainty of infective endocarditis (IE) diagnosis	SMR01 diagnostic code position 1, n (%)	SMR01 diagnostic code positions 1 and 2, n (%)
True IE	202/208 (97.1%)	234/264 (88.6%)
No IE	6/208 (2.8%)	30/264 (11.4%)

Supplementary text IV: Description of 5 year lookback period and calculation of person-time

Lookback

The schematic below demonstrates an example of how the 5-year lookback period was employed in the period between 2000 and 2015 (inclusive) to identify incident events of infective endocarditis in three exemplar patients (patients A, B and C). The total incident count for each year is shown in the final column. Where a patient has been admitted with an episode of infective endocarditis, a '1' appears in the 'Admission' column. If no infective endocarditis event has occurred in the 5-years prior (i.e. the 'lookback' period, as indicated by the light grey shading), then the event is considered an incident event and a '1' will also appear in the 'Incident' column.

Year	Patient A		Patient B		Patient C		Total incident events
	Admission	Incident	Admission	Incident	Admission	Incident	
**2000**	0	0	0	0	0	0	**0**
**2001**	0	0	0	0	0	0	**0**
**2002**	0	0	0	0	0	0	**0**
**2003**	0	0	0	0	0	0	**0**
**2004**	0	0	0	0	0	0	**0**
**2005**	1	1	0	0	1	1	**2**
**2006**	1	0	1	1	0	0	**1**
**2007**	0	0	0	0	1	0	**0**
**2008**	0	0	0	0	0	0	**0**
**2009**	1	0	0	0	0	0	**0**
**2010**	0	0	0	0	0	0	**0**
**2011**	0	0	0	0	0	0	**0**
**2012**	0	0	0	0	0	0	**0**
**2013**	0	0	0	0	1	1	**1**
**2014**	0	0	0	0	0	0	**0**
**2015**	1	1	1	1	1	0	**2**

Person-time calculation

The schematic below demonstrates an example across three patients on how the person-time was calculated.

**Year**	**POP**	**P1**	**P2**	**P3**	**PT**
2004	190	0	1	1	189
2005	190	0	1	1	189
2006	190	0	1	0	188
2007	190	0	1	0	188
2008	190	0	1	0	188
2009	190	1	1	0	189
2010	185	1	1	0	184
2011	185	1	0	1	184
2012	185	1	0	1	184
2013	185	1	0	1	184
2014	185	1	0	1	184

POP refers to the mid-year estimate for the population (based on National Records Scotland census data and mid-year estimation modelling). P1, P2 and P3 refers to the person-time for each of the 3 patients with incident infective endocarditis. Patient 1 had an admission in 2004, they were not eligible to have another incident event within 5 years, and so the person-time for each of these periods is removed. Patient 2 had an incident event in 2011 and so only contributed 7 person-years. Patient 3 had an event in 2006 which was not incident, and as a consequence did not contribute to the period from 2006 to 2010 (inclusive).

The person-time for each year, within each stratum, is therefore calculated as follows:
$PT = POP - N + p1 + p2 + p3 + ...pn$ where N refers to the total number of individuals with incident infective endocarditis (in the above example, N would be equal to 3 as there are 3 patients).

Supplementary text V: Use of ATC coding to determine comorbidity status

**Comorbidity**	**Relevant class of drug (British National Formulary 62)**	**Chapter and section in the British National Formulary**
Chronic respiratory disease	Respiratory system: all drugs	Chapter 3
Diabetes mellitus	Endocrine system: drugs used in diabetes	Chapter 6, section 6.1

Supplementary text VI: Details of interrupted time series analysis

To evaluate any change in the incidence of infective endocarditis before and after introduction of guidelines on antibiotic prophylaxis published by the National Institute of Health and Care Excellence, an interrupted time series analysis model was created.

We chose a step-change model. A Poisson model was used as we were predicting count data. We modelled the count data directly (rather than the rate which doesn't follow a Poisson distribution), using the person-time (log transformed) as an offset variable in order to transform back to rates. In order to account for overdispersion we used a quasipoisson model (allowing the variance to be proportional rather than equal to the mean).

The following model was created with corresponding output:

model <- glm(count ~ offset(log(person_time)) + guidelines + year + guidelines*year, family=quasi-poisson, data)

Term	estimate	std.error	statistic	p-value
**(Intercept)**	-6.7862	25.2561	-0.2687	0.794
**Guidelines**	0.3997	34.9581	0.0114	0.991
**Year**	-0.0014	0.0126	-0.1082	0.916
**Guidelines*Year**	-0.0002	0.0174	-0.0098	0.992

The plot below shows no auto correlation removing the linear dependence of the lags using the partial autocorrelation function in the stats package in R Version 3·5·1 (Vienna, Austria):

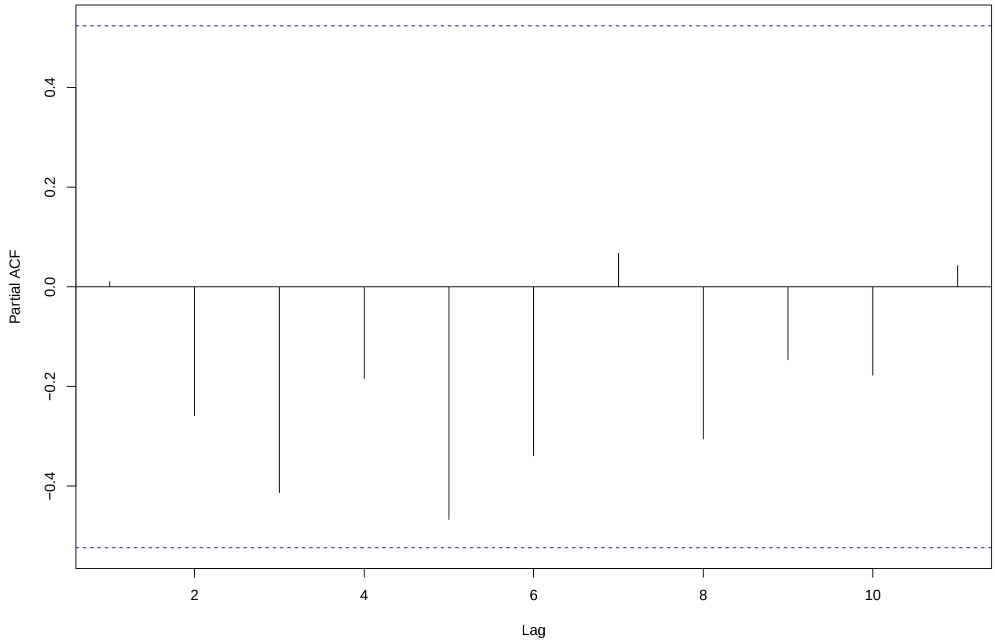

Supplementary tables

Supplementary table I: Baseline characteristics and short- and long-term outcomes stratified by single calendar years from 2008 to 2014

	Single-year groups						
	**2008**	**2009**	**2010**	**2011**	**2012**	**2013**	**2014**
**Number of patients, n**	**340**	**311**	**317**	**312**	**315**	**311**	**361**
**Age, years**	66.2 (17.0)	66.8 (16.9)	65.9 (17.6)	67.5 (18.4)	66.5 (17.1)	67.7 (18.2)	65.4 (18.5)
**Women, n (%)**	167 (49.1)	142 (45.7)	144 (45.4)	151 (48.4)	151 (47.9)	160 (51.4)	162 (44.9)
**Scottish index of multiple deprivation (SIMD) index, n (%)**							
Rank 1 (most deprived)	80 (23.5)	82 (26.5)	72 (22.7)	69 (22.4)	66 (21.2)	72 (23.3)	86 (24.0)
Rank 2	71 (20.9)	73 (23.5)	65 (20.5)	79 (25.6)	82 (26.3)	72 (23.3)	78 (21.7)
Rank 3	77 (22.6)	61 (19.7)	68 (21.5)	64 (20.8)	59 (18.9)	50 (16.2)	78 (21.7)
Rank 4	62 (18.2)	50 (16.1)	64 (20.2)	47 (15.3)	47 (15.1)	58 (18.8)	58 (16.2)
Rank 5 (least deprived)	50 (14.7)	44 (14.2)	48 (15.1)	49 (15.9)	58 (18.6)	57 (18.4)	59 (16.4)
**Previous medical conditions / interventions**							
Myocardial infarction, n (%)	15 (4.4)	18 (5.8)	16 (5.0)	21 (6.7)	14 (4.4)	14 (4.5)	15 (4.2)
Cerebrovascular disease, n (%)	18 (5.3)	19 (6.1)	16 (5.0)	15 (4.8)	14 (3.4)	17 (5.5)	13 (3.6)
Heart failure hospitalization, n (%)	39 (11.5)	46 (14.8)	35 (11.0)	45 (14.4)	31 (9.8)	32 (10.3)	30 (8.3)
Cardiac device, n (%)	< 5	6 (1.9)	10 (3.2)	10 (3.2)	9 (2.9)	8 (2.6)	11 (3.0)
Cardiac valvular surgery, n (%)	15 (4.4)	26 (8.4)	35 (11.0)	29 (9.3)	22 (7.0)	22 (7.1)	22 (6.1)
Chronic respiratory disease, n (%)	-	42 (23.1)	69 (21.8)	51 (16.3)	56 (17.8)	51 (16.4)	65 (18.0)
Diabetes mellitus, n (%)	-	26 (14.3)	38 (12.0)	39 (12.5)	50 (15.9)	41 (13.2)	55 (15.2)
**Outcomes at 30 days**							
All-cause death, n (%)	48 (14.1)	30 (9.6)	51 (16.1)	54 (17.3)	39 (12.4)	32 (10.3)	47 (13.0)
Heart failure hospitalization, n (%)	20 (5.9)	20 (6.4)	18 (5.7)	14 (4.5)	13 (4.1)	17 (5.5)	12 (3.3)
Valve surgery, n (%)	20 (5.9)	15 (4.8)	20 (6.3)	15 (4.8)	12 (3.8)	17 (5.5)	22 (6.1)
**Outcomes at 1 year**							
All-cause death, n (%)	114 (33.5)	84 (27.0)	99 (31.2)	96 (30.8)	97 (30.8)	98 (31.5)	110 (30.5)
Heart failure hospitalization, n (%)	42 (12.4)	42 (13.5)	35 (11.0)	35 (11.2)	33 (10.5)	29 (9.3)	26 (7.2)
Valve surgery, n (%)	48 (14.1)	43 (13.8)	49 (15.5)	31 (9.9)	29 (9.2)	34 (10.9)	38 (10.5)

Supplementary table II: Count data and estimated incidence rate per 100,000 stratified by year and sex

Year	Number of cases, n			Estimated crude incidence rate per 100,000								
	Overall	Males	Females	Overall	Lower 95% CI	Upper 95% CI	Males	Lower 95% CI	Upper 95% CI	Females	Lower 95% CI	Upper 95% CI
**1990**	201	100	101	5.34	4.80	5.94	5.62	4.87	6.48	5.28	4.54	6.15
**1991**	225	106	119	6.46	6.05	6.89	6.26	5.71	6.87	6.62	6.03	7.28
**1992**	318	130	188	7.53	7.08	8.02	6.91	6.34	7.53	7.95	7.29	8.66
**1993**	305	135	170	8.28	7.80	8.79	7.49	6.90	8.14	8.84	8.14	9.59
**1994**	330	137	193	8.59	8.13	9.08	7.98	7.39	8.63	9.09	8.41	9.82
**1995**	333	165	168	8.63	8.14	9.14	8.37	7.75	9.05	8.90	8.22	9.64
**1996**	326	143	183	8.58	8.11	9.07	8.63	8.01	9.31	8.59	7.94	9.29
**1997**	314	156	158	8.51	8.05	9.00	8.71	8.08	9.38	8.37	7.73	9.06
**1998**	326	164	162	8.40	7.93	8.90	8.55	7.92	9.24	8.25	7.60	8.95
**1999**	330	152	178	8.21	7.76	8.68	8.24	7.63	8.89	8.15	7.52	8.82
**2000**	291	130	161	7.94	7.49	8.41	7.87	7.28	8.51	7.98	7.35	8.66
**2001**	291	138	153	7.66	7.22	8.13	7.56	6.99	8.19	7.75	7.13	8.42
**2002**	278	131	147	7.41	6.99	7.86	7.35	6.79	7.95	7.48	6.88	8.12
**2003**	303	144	159	7.22	6.80	7.67	7.24	6.68	7.84	7.23	6.64	7.88
**2004**	272	130	142	7.11	6.70	7.56	7.24	6.68	7.84	7.06	6.48	7.69
**2005**	264	119	145	7.14	6.73	7.57	7.41	6.86	8.01	6.97	6.41	7.59
**2006**	282	153	129	7.32	6.89	7.77	7.77	7.19	8.40	6.96	6.38	7.59
**2007**	314	160	154	7.60	7.18	8.06	8.24	7.65	8.88	6.98	6.42	7.60
**2008**	332	169	163	7.83	7.40	8.29	8.61	8.00	9.27	6.99	6.42	7.61
**2009**	302	164	138	7.84	7.39	8.31	8.66	8.04	9.33	6.95	6.37	7.58
**2010**	314	171	143	7.61	7.20	8.05	8.40	7.81	9.03	6.87	6.32	7.47
**2011**	302	155	147	7.36	6.94	7.81	8.08	7.49	8.72	6.82	6.25	7.44
**2012**	307	160	147	7.32	6.89	7.78	8.00	7.40	8.64	6.87	6.29	7.51
**2013**	302	144	158	7.60	7.19	8.05	8.29	7.69	8.94	7.05	6.47	7.68
**2014**	351	193	158	8.14	7.47	8.87	8.88	7.93	9.94	7.30	6.41	8.31

Supplementary table III: Count data and estimated incidence rate per 100,000 stratified by year and age group

	Estimated crude incidence rate per 100,000											
	20-39 years			40-59 years			60-79 years			≥80 years		
Year	Rate per 100,000	Lower 95% CI	Upper 95% CI	Rate per 100,000	Lower 95% CI	Upper 95% CI	Rate per 100,000	Lower 95% CI	Upper 95% CI	Rate per 100,000	Lower 95% CI	Upper 95% CI
**1990**	2.27	2.04	2.51	4.70	3.93	5.63	11.50	10.01	13.21	17.66	13.36	23.32
**1991**	2.27	2.06	2.50	5.03	4.46	5.69	13.56	12.37	14.87	20.06	16.22	24.80
**1992**	2.28	2.08	2.49	5.35	4.80	5.96	15.78	14.59	17.08	22.55	18.99	26.78
**1993**	2.28	2.10	2.48	5.58	5.02	6.20	17.87	16.58	19.25	24.89	21.40	28.94
**1994**	2.29	2.11	2.47	5.67	5.11	6.28	19.48	18.14	20.91	26.90	23.35	30.98
**1995**	2.29	2.13	2.47	5.57	5.02	6.18	20.39	19.00	21.88	28.56	24.89	32.76
**1996**	2.30	2.14	2.46	5.33	4.81	5.91	20.56	19.19	22.02	29.93	26.17	34.22
**1997**	2.30	2.16	2.45	5.02	4.52	5.57	20.10	18.76	21.54	31.03	27.20	35.40
**1998**	2.31	2.17	2.45	4.72	4.24	5.25	19.21	17.90	20.62	31.85	27.97	36.26
**1999**	2.31	2.18	2.45	4.46	4.01	4.97	18.09	16.85	19.42	32.34	28.47	36.74
**2000**	2.32	2.19	2.45	4.27	3.82	4.76	16.94	15.74	18.22	32.54	28.69	36.92
**2001**	2.32	2.20	2.45	4.10	3.67	4.58	15.92	14.77	17.16	32.52	28.70	36.84
**2002**	2.33	2.20	2.45	3.96	3.55	4.43	15.17	14.06	16.35	32.35	28.59	36.59
**2003**	2.33	2.21	2.46	3.86	3.45	4.32	14.74	13.65	15.92	32.09	28.38	36.29
**2004**	2.34	2.21	2.47	3.81	3.41	4.26	14.66	13.58	15.82	31.82	28.16	35.95
**2005**	2.34	2.21	2.48	3.83	3.43	4.27	14.84	13.76	16.00	31.63	28.02	35.71
**2006**	2.35	2.20	2.50	3.89	3.49	4.35	15.13	14.03	16.32	31.65	28.04	35.73
**2007**	2.35	2.20	2.51	3.97	3.56	4.42	15.34	14.24	16.53	31.94	28.32	36.02
**2008**	2.36	2.19	2.53	3.99	3.58	4.45	15.28	14.19	16.46	32.48	28.82	36.60
**2009**	2.36	2.19	2.55	3.95	3.54	4.41	14.91	13.82	16.07	33.18	29.46	37.37
**2010**	2.37	2.18	2.57	3.89	3.49	4.34	14.31	13.27	15.43	33.96	30.22	38.15
**2011**	2.37	2.17	2.59	3.89	3.48	4.34	13.68	12.66	14.79	34.78	31.00	39.03
**2012**	2.38	2.17	2.61	4.00	3.58	4.46	13.17	12.17	14.25	35.69	31.72	40.17
**2013**	2.38	2.16	2.63	4.24	3.78	4.77	12.82	11.77	13.97	36.72	32.06	42.07
**2014**	2.39	2.15	2.65	4.59	3.88	5.43	12.58	11.09	14.27	37.85	31.51	45.46

Supplementary table IV: Model coefficients from generalized additive model evaluating all-cause mortality by calendar year, adjusted for age and comorbidity

	**Estimate**	**Standard error**	**z value**	**Odds ratio**	**Lower 95% CI**	**Upper 95% CI**	**p-value**
**(Intercept)**	-0.50	0.10	-4.90	-	-	-	-
**Female sex**	-0.16	0.05	-3.03	0.85	0.75	0.96	0.002
**Deprivation (per unit increment)**	-0.09	0.02	-4.69	0.92	0.88	0.95	<0.001
**Heart failure hospitalization**	0.74	0.07	10.75	2.09	1.96	2.22	<0.001
**Myocardial infarction**	0.08	0.12	0.66	1.08	0.85	1.31	0.512
**Cerebrovascular disease**	0.25	0.11	2.27	1.28	1.07	1.49	0.023

**Estimates of non-linear smooth functions**	**Estimated degrees of freedom**	**Chi-squared**	**p-value**
**s(year)**	1.01	4.48	0.035
**s(age_years)**	5.64	486.50	<0.001

Supplementary table V: Predicted one-year mortality in women and men from the generalized additive models

YEAR	WOMEN			MEN		
	**Probability**	**95% LL**	**95% UL**	**Probability**	**95% LL**	**95% UL**
**1990**	27.32	24.62	30.21	30.66	27.71	33.78
**1991**	27.17	24.54	29.96	30.50	27.64	33.51
**1992**	27.01	24.46	29.72	30.33	27.57	33.24
**1993**	26.86	24.38	29.48	30.16	27.48	32.98
**1994**	26.70	24.29	29.26	29.99	27.39	32.73
**1995**	26.55	24.19	29.04	29.83	27.30	32.49
**1996**	26.39	24.09	28.83	29.66	27.20	32.26
**1997**	26.24	23.98	28.63	29.50	27.09	32.03
**1998**	26.09	23.86	28.44	29.33	26.97	31.82
**1999**	25.93	23.73	28.26	29.17	26.84	31.61
**2000**	25.78	23.60	28.09	29.01	26.71	31.42
**2001**	25.63	23.45	27.93	28.85	26.56	31.24
**2002**	25.48	23.31	27.78	28.68	26.41	31.07
**2003**	25.33	23.15	27.64	28.52	26.25	30.90
**2004**	25.18	22.99	27.51	28.36	26.08	30.75
**2005**	25.03	22.82	27.39	28.20	25.91	30.61
**2006**	24.88	22.64	27.27	28.04	25.72	30.48
**2007**	24.74	22.46	27.17	27.88	25.53	30.36
**2008**	24.59	22.27	27.07	27.72	25.33	30.24
**2009**	24.44	22.08	26.97	27.56	25.13	30.14
**2010**	24.30	21.88	26.89	27.40	24.92	30.04
**2011**	24.15	21.68	26.81	27.25	24.70	29.95
**2012**	24.01	21.47	26.73	27.09	24.49	29.86
**2013**	23.86	21.27	26.66	26.93	24.26	29.79
**2014**	23.72	21.06	26.60	26.78	24.03	29.71

Supplementary table VI: Logistic regression model coefficients and standard errors with mortality at 30 days as the primary outcome.

	**Estimate**	**Std. Error**	**z value**	**Pr (>|z|)**
(Intercept)	-2.66	0.31	-8.57	<0.001
**Baseline characteristics**				
Age	0.02	0.01	1.31	0.191
Sex	0.08	0.15	0.51	0.612
Deprivation (per unit increment)	0.02	0.05	0.36	0.720
Age:sex interaction	<0.001	0.009	0.08	0.936
**Co-morbidities**				
Heart failure hospitalization	0.59	0.19	3.05	0.002
Myocardial infarction	0.33	0.28	1.18	0.238
Cerebrovascular disease	0.18	0.30	0.60	0.546
Chronic lung disease	0.13	0.18	0.72	0.470
Diabetes mellitus	-0.21	0.21	-0.98	0.328
**Microbiology**				
Enterococcus sp.	0.65	0.35	1.86	0.06
Polymicrobial/other	0.24	0.36	0.66	0.511
Staphylococcus aureus	1.40	0.20	7.17	<0.001
Coagulase negative staphylococci	1.26	0.28	4.45	<0.001
Streptococcus sp.	0.65	0.21	3.15	0.002

Supplementary table VII: Logistic regression model coefficients and standard errors with mortality at 1 year as the primary outcome.

	Estimate	Std. Error	z value	Pr (>|z|)
(Intercept)	-1.18	0.23	-5.04	<0.001
**Baseline characteristics**				
Age (per 10 years)	0.04	0.01	3.69	<0.001
Sex	-0.14	0.12	-1.16	0.247
Deprivation (per unit increment)	-0.04	0.04	-1.08	0.282
Age:sex interaction	-0.001	0.007	-0.14	0.889
**Co-morbidities**				
Heart failure hospitalization	0.87	0.16	5.38	<0.001
Myocardial infarction	0.42	0.23	1.82	0.069
Cerebrovascular disease	-0.21	0.25	-0.81	0.418
Chronic lung disease	0.04	0.14	0.30	0.765
Diabetes mellitus	0.07	0.16	0.43	0.671
**Microbiology**				
Enterococcus sp.	1.23	0.26	4.70	<0.001
Polymicrobial/other	0.71	0.25	2.89	0.004
Staphylococcus aureus	1.47	0.17	8.69	<0.001
Coagulase negative staphylococci	1.03	0.25	4.15	<0.001
Streptococcus sp.	0.51	0.16	3.10	0.002
